# Pneumonitis in Combined Anti-programmed Death-1 Immunotherapy and Radiation Therapy for Renal Cell Carcinoma

**DOI:** 10.7759/cureus.3748

**Published:** 2018-12-18

**Authors:** Nathan Y Yu, Michael Deftos, Clifford C Wang

**Affiliations:** 1 Radiation Oncology, Santa Clara Valley Medical Center, San Jose, USA; 2 Pathology, Santa Clara Valley Medical Center, San Jose, USA; 3 Internal Medicine, Santa Clara Valley Medical Center, San Jose, USA

**Keywords:** immunotherapy, pneumonitis, radiation therapy, nivolumab, renal cell carcinoma

## Abstract

Anti-programed cell death-1 (Anti-PD-1) is a promising immunotherapy for advanced cancers. Autoimmune pneumonitis is a rare but potentially serious toxicity induced by anti-PD-1 immunotherapy. We report a case of therapy-induced pneumonitis in the setting of combined nivolumab, anti-PD-1 immunotherapy, and radiation therapy for metastatic renal cell carcinoma (RCC).

## Introduction

The human immune system has been shown to be able to mount an immune response capable of recognizing and eliminating cancer cells. However, tumors have been able to persist despite a host immune response, implying a mechanism of evasion. Adaptive immune resistance describes a mechanism by which cancer cells change their phenotype to suppress the host immune response [1-3]. Anti-programmed death-1 (PD-1) immunotherapy is promising for advanced cancers [4]. Anti-PD-1 immunotherapy inhibits tumor cells from evading the host immune response by preventing programmed death ligand-1 expressed on tumor cells from interacting with PD-1 receptors on immune cells [5]. As a result, PD-1 receptor interaction negatively regulates T cell activity, thus inhibiting tumor immunity [5]. Nivolumab is an anti-PD-1 immunotherapy that has been approved for multiple advanced cancers including renal cell carcinoma (RCC) [6]. Autoimmune pneumonitis is a rare but serious potential toxic effect of PD-1 immune checkpoint blockade [7]. Here we describe nivolumab-induced pneumonitis in a 60-year-old male with metastatic RCC in the setting of combined nivolumab and radiation therapy.

## Case presentation

**Figure 1:**
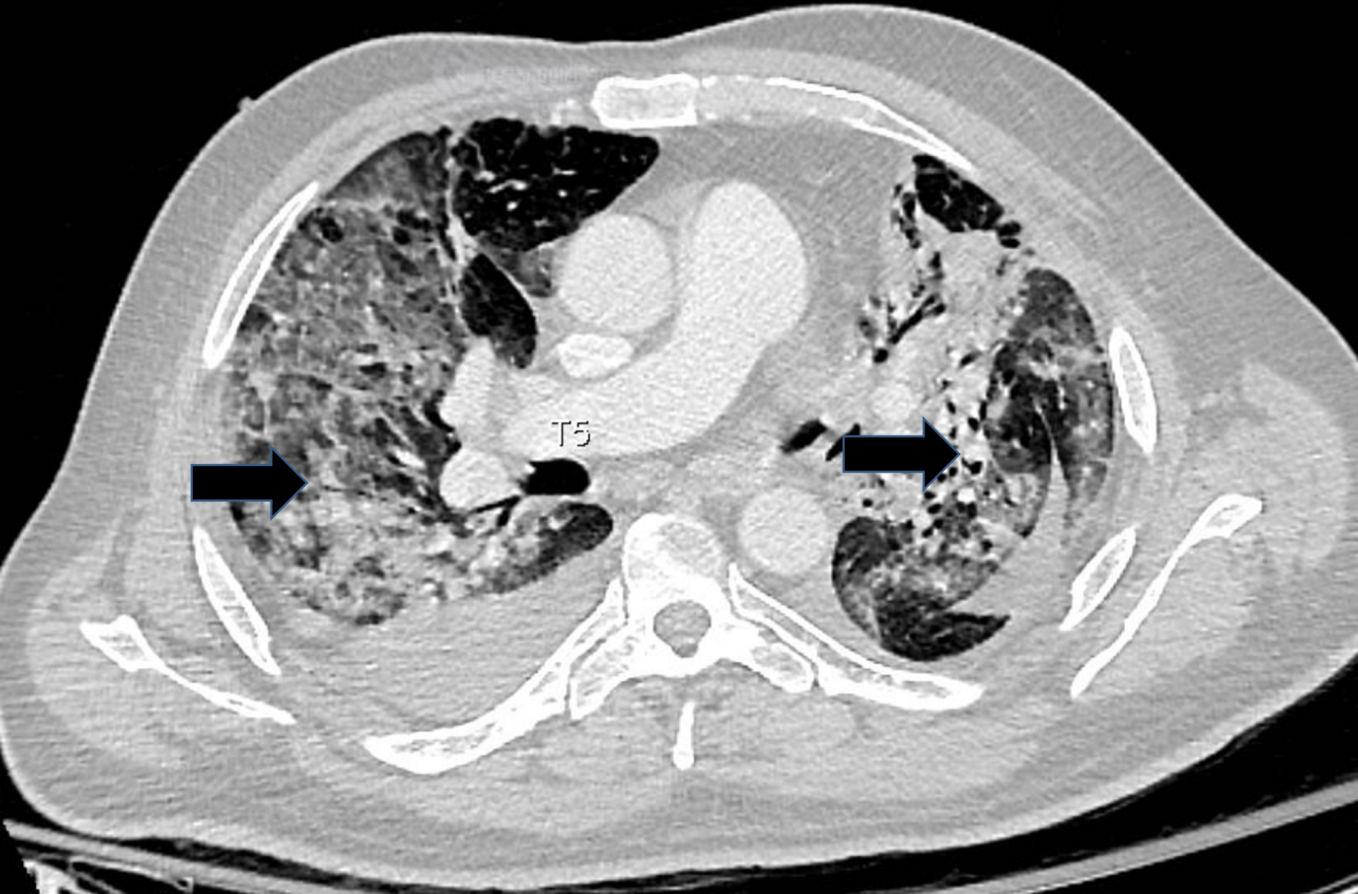
Computed Tomography of the Chest (Axial View). Bilateral diffuse areas of ground-glass opacities involving the majority of the right and left lung consistent with interstitial pneumonitis.

**Figure 2:**
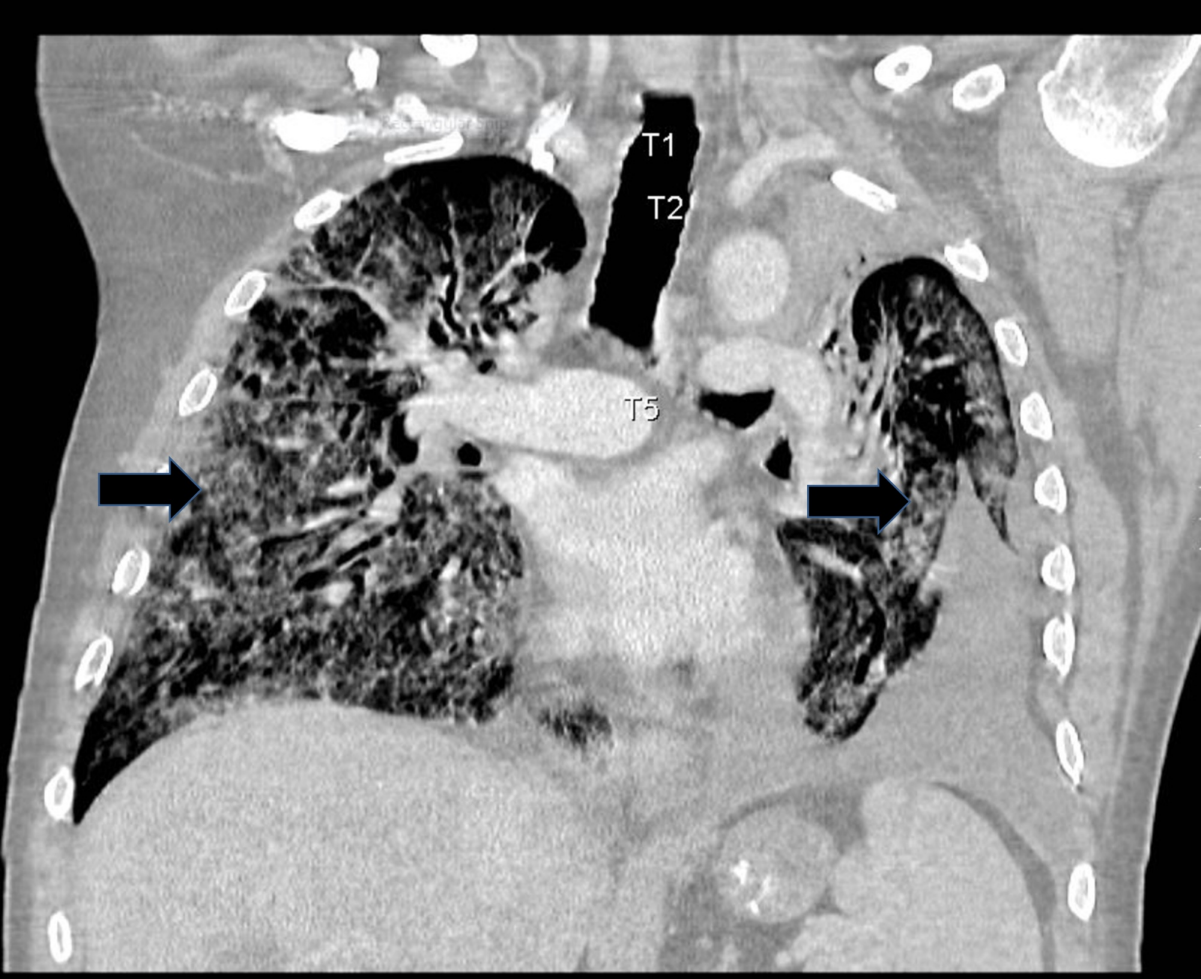
Computed Tomography of the Chest (Coronal View). Bilateral diffuse areas of ground-glass opacities involving the majority of the right and left lung consistent with interstitial pneumonitis.

A 60-year-old male with metastatic RCC treated with nivolumab and palliative radiation therapy presented to our institution in 2016 with shortness of breath and was found to be in acute respiratory failure. Computed tomography (CT) of the chest was significant for multiple new ground-glass opacities throughout bilateral lungs concerning for therapy-induced pneumonitis (Figures 1, 2). The etiology of ground glass opacities includes but is not limited to infectious pneumonitis, bronchioloalveolar carcinoma, or interstitial disease. Given the timing of symptom onset as well as lack of response to infectious treatment, therapy-induced pneumonitis remained high on our differential.

He initially presented in 2011 with gross hematuria and right-sided flank pain and underwent right radical nephrectomy and lymph node dissection of a 9 cm Fuhrman grade IV RCC with negative margins and lymph nodes. Two years later, surveillance imaging and biopsy were significant for metastatic RCC in the lungs. He was initially treated with one year of sunitinib, a multi-targeted receptor tyrosine kinase inhibitor. However, given the progression of disease, he was transitioned to one year of pazopanib followed by six months of axitinib, one month of everolimus, and five months of sorafenib. Pazopanib, axitinib, and sorafenib are also tyrosine kinase inhibitors. Everolimus is an inhibitor of mammalian target of rapamycin. Given the lack of response to these therapies, our patient was started on nivolumab at 3 mg/kg in May of 2016. Over the course of four years, he received targeted palliative radiotherapy including 1900 centigray (cGy) to a left upper lobe lung mass in May 2016 and 800 cGy to an L5 lesion in September 2016.

He complained of chronic shortness of breath for three months felt secondary to anemia and a left pleural effusion before presenting to our hospital in acute respiratory failure with CT evidence of new diffuse ground-glass opacities occupying the majority of both lungs (Figures 1, 2). Given high suspicion for therapy-induced pneumonitis, he was started on a treatment course of high dose steroids. However, the patient’s respiratory status continued to decline and he passed away on comfort measures.

**Figure 3:**
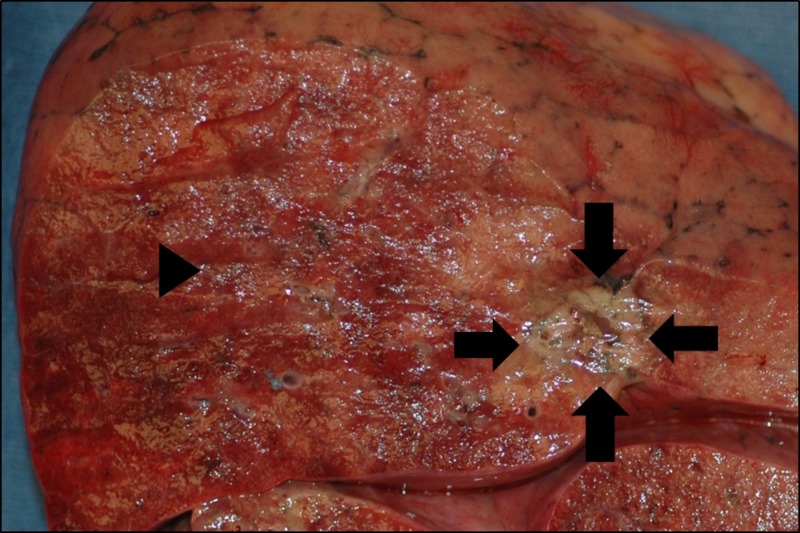
Gross Appearance of Cut Surface of Left Lung. The arrows indicate a 1.5 cm tumor of metastatic renal cell carcinoma in the upper lobe. The arrowhead shows adjacent hemorrhagic lung parenchyma.

**Figure 4:**
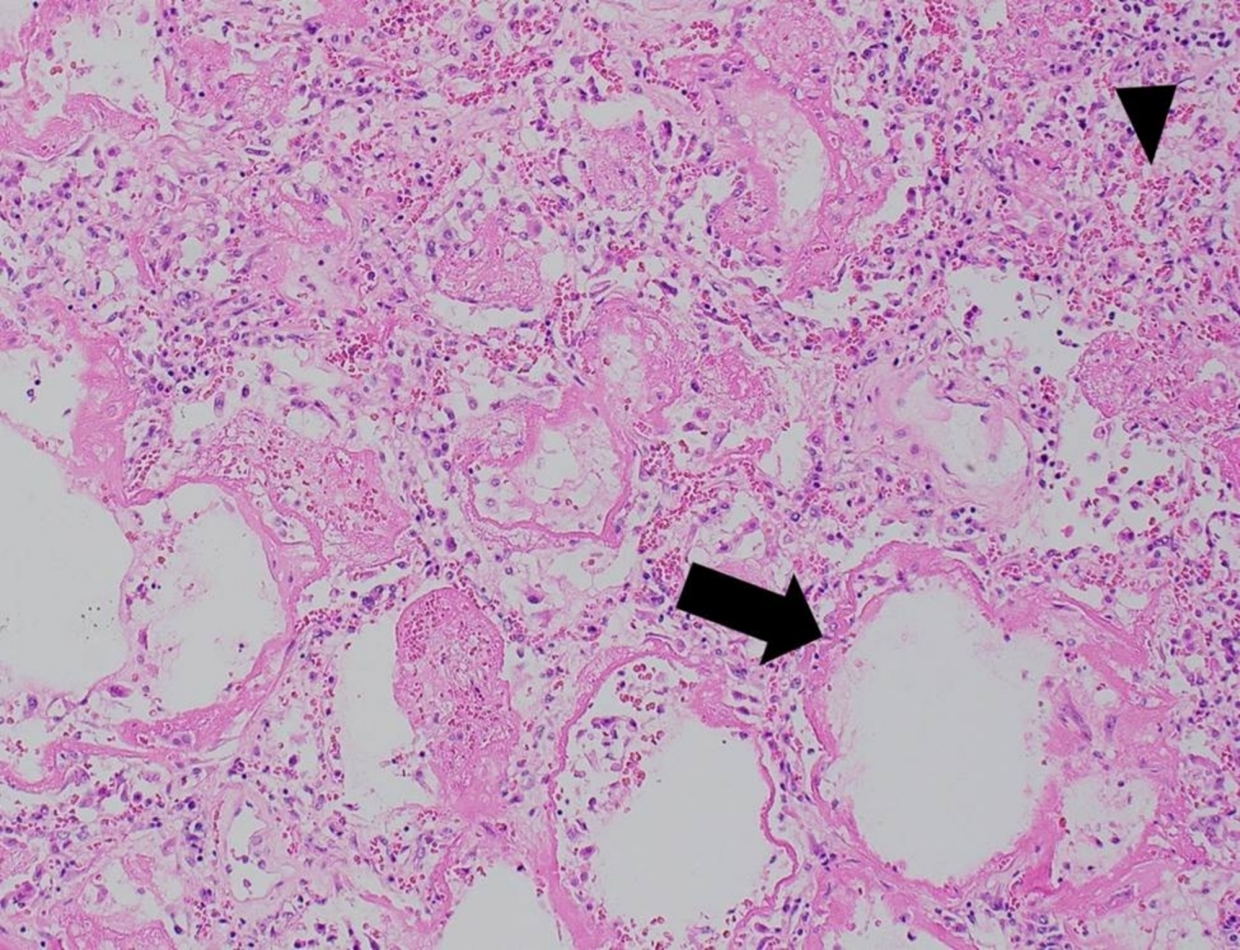
Histologic Appearance of Lung Parenchyma. Hematoxylin and eosin stained histologic section of lung parenchyma at 20x magnification. Arrow shows eosinophilic hyaline membrane lining alveolar septa. Arrow head shows lung interstitium with hemorrhage, proteinaceous exudate and scattered inflammatory cells. These features are consistent with a diffuse alveolar damage pattern of lung injury.

Pathology was significant for organizing diffuse alveolar damage with hyaline membrane formation in all lobes of both lungs away from the metastatic RCC (Figures 3, 4). There was no evidence of an infectious process from cultures and pathologic evaluation. This histologic reaction pattern is a typical finding in patients with a clinical diagnosis of acute respiratory distress syndrome (ARDS) concerning for therapy-induced pneumonitis.

## Discussion

The human immune system has been shown to be able to mount an immune response capable of recognizing and eliminating cancer cells. However, tumors have been able to persist despite a host immune response, implying a mechanism of evasion. Adaptive immune resistance describes a mechanism by which cancer cells change their phenotype to suppress the host immune response [1-3].

Immune checkpoints are critical for maintaining self-tolerance and immune homeostasis. Under normal circumstances, the programmed death-ligand pathway is an immune checkpoint that is essential for regulating the host immune response. PD-1 is an inhibitory receptor of the Ig superfamily expressed on activated T cells. When programmed death-ligand 1 (PD-L1) interacts with PD-1 on activated T cells, inhibitory signals are transmitted that reduce cytotoxic T-cell proliferation. The programmed death-ligand pathway downregulates self-reactive T cells and prevents autoimmune disease [8].

Cancer cells hijack the programmed death-ligand self-tolerance pathway and evade immune surveillance by overexpressing PD-L1 thus increasing ligation with PD-1 on activated T cells [1-3,8]. Emerging therapies that are directed at blockade of PD-1 have shown promising results [1,9]. Nivolumab is a human IgG4 anti-PD-1 monoclonal antibody that prevents cancer cells from suppressing activated T cells. It is currently approved by the United States Food and Drug Administration for the treatment of various cancers including melanoma, non-small cell lung cancer, and RCC. Of particular relevance to our case, nivolumab has shown a promising role in metastatic RCC [10].

Our patient represented with metastatic RCC after two years without evidence of recurrent disease. He was treated with multiple rounds of chemotherapy including small-molecule, multi-targeted receptor tyrosine kinase inhibitors, and a mammalian target of rapamycin inhibitor. Given the progression of disease, the patient was transitioned to combined nivolumab, an anti-PD-1 immunotherapy, and palliative radiation therapy. After four months of combined therapy, the patient passed away from acute respiratory failure secondary to therapy-induced pneumonitis.

Anti-PD-1 immunotherapy aims to revitalize a suppressed immune response to foreign cancer cells. Multiple studies and clinical trials of nivolumab for various cancers have shown promising results [10-14]. In particular, patients with advanced treatment-refractory RCC have demonstrated durable responses [10,13,14]. However, blockade of the PD-ligand pathway can lead to unique toxicities.

Nivolumab-induced pneumonitis is a rare but clinically significant toxicity that has been previously reported [7,8,14]. The median time from induction of therapy to nivolumab-induced pneumonitis is reported to be approximately two to three months [8]. The risk has been quoted to be about one percent [14]. The odds of developing therapy-induced pneumonitis in patients with advanced cancer are much higher with combination anti-PD-1 therapy than with monotherapy [15]. Radiographic characteristics of nivolumab-induced pneumonitis include diffuse ground glass and reticular opacities [8]. Therapy-induced pneumonitis is a serious toxicity especially in patients with baseline poor lung reserve secondary to metastatic disease in the lung.

Studies of combination therapy with targeted radiation and anti-PD-1 immunotherapy have been exciting and promising. Previous studies have suggested that radiation therapy can induce an abscopal effect in nonirradiated lesions [16]. The current rationale is that radiation-induced release of antigens from tumor cells within a radiation treatment field stimulates an enhanced antitumor immune response that has an effect throughout the body. The synergistic effect of combination immunotherapy and radiation therapy has been suggested to improve outcomes. However, the risk of anti-PD-1 toxicity may be increased in the setting of concurrent radiation therapy. Combined palliative radiation therapy and nivolumab may have increased our patient’s risk for development of therapy-induced pneumonitis.

Treatment of nivolumab-induced pneumonitis involves holding anti-PD-1 therapy and immunosuppression. Immunosuppressive regimens most commonly include corticosteroids (85%), with or without the addition of infliximab (15%) [8]. After completion of a corticosteroid taper without nivolumab treatment, patients can restart anti-PD-1 therapy but are at risk for recurrent pneumonitis.

## Conclusions

Treatment of cancer with anti-PD-1 is an emerging immunotherapy with promising outcomes. Pneumonitis is a rare but clinically significant potential toxic effect that can evolve rapidly. Our case highlights the risk of developing therapy-induced pneumonitis with anti-PD-1 immunotherapy and suggests that the addition of concurrent radiation therapy may further increase this risk. Close monitoring during treatment is essential in all patients receiving both monotherapy and combined immunotherapy and radiation therapy.
